# Unraveling the Genome-Wide Impact of Recombinant Baculovirus Infection in Mammalian Cells for Gene Delivery

**DOI:** 10.3390/genes11111306

**Published:** 2020-11-04

**Authors:** Ha Youn Shin, Hanul Choi, Nahyun Kim, Nayoung Park, Heesun Kim, Jaebum Kim, Young Bong Kim

**Affiliations:** 1 Department of Biomedical Science & Engineering, Konkuk University, Seoul 05029, Korea; hayounshin@konkuk.ac.kr (H.Y.S.); knh64@naver.com (N.K.); p3159@konkuk.ac.kr (N.P.); kimheesun2@konkuk.ac.kr (H.K.); 2 Department of Bio-Industrial Technologies, Konkuk University, Seoul 05029, Korea; chlgksmf9977@hanmail.net

**Keywords:** recombinant baculovirus, total RNA-seq, viral gene expression, immune response

## Abstract

Baculovirus expression systems have been widely used to produce recombinant mammalian proteins owing to the lack of viral replication in vertebrates. Although several lines of evidence have demonstrated impacts of baculovirus infection in mammalian hosts, genome-wide effects have not been fully elucidated. Here, we provide comparative transcriptome profiles of baculovirus and host-immune response genes in recombinant baculovirus-infected mammalian and insect cells. Specifically, to decipher the impacts of baculovirus infection in mammalian cells, we conducted total RNA-seq on human 293TT cells and insect Sf9 cells infected with recombinant baculovirus. We found that baculovirus genes were rarely expressed under the control of baculoviral promoters in 293TT cells. Although some baculovirus early genes, such as *PE38* and *IE-01*, showed limited expression in 293TT cells, baculoviral late genes were mostly silent. We also found modest induction of a small number of mammalian immune response genes associated with Toll-like receptors, cytokine signaling, and complement in baculovirus-infected 293TT cells. These comprehensive transcriptome data will contribute to improving recombinant baculovirus as tools for gene delivery, gene therapy, and vaccine development.

## 1. Introduction

Baculoviruses have been extensively used for decades as eukaryotic gene expression systems. Although baculoviruses primarily use insects as a host to produce viral progenies, they are also capable of infecting some mammalian cells, despite deficiencies in their ability to replicate in these cells [1]. This unique feature allows baculoviruses to serve as potent and safe mammalian gene-delivery systems [2,3]. Baculovirus expression systems offer some additional benefits compared with other currently used gene expression systems. For example, unlike bacterial expression systems, baculovirus systems support post-translational modifications, enabling production of mammalian proteins in their native form [4]. Compared with adeno-associated virus (AAV) vectors, which have a limited gene insertion capacity (~4.7 kb), baculoviruses can accept large (>100 kbp) foreign DNA fragments [2]. Finally, because baculoviruses do not need any helper viruses for replication, producing recombinant viruses is relatively fast and easy.

One of the most widely used baculoviruses for gene expression systems is *Autographa californica* multiple nuclear polyhedrosis virus (AcMNPV), a member of the *Baculoviridae* family [3]. Baculoviruses commonly have large (80–200 kbp), circular, double-stranded DNA genomes containing over 100 genes. Baculovirus gene expression is regulated in a phase-dependent manner following virus infection [3,5]. Baculoviruses are known to enter insect cells by endocytosis through fusion of the viral surface protein GP64 with the endosomal membrane at low pH [6]. Following virus entry into cells, immediate early genes, including *IE-1* and its splice variant *IE-01*, are expressed in host cells [7,8]. Later, late expression factors (*lefs*), including *lef1* through *lef12*, are expressed in host cells, followed by the expression of very late genes, such as *p10* and *Vlf-1* [9,10]. Several studies have also reported that baculovirus infection of mammals can result in induction of some immune response genes [11,12,13,14,15,16,17]. For example, expression levels of Toll-like receptor, proinflammatory cytokines, and complement factors are elevated in baculovirus-infected mammalian cells and mice. 

Although several studies have reported physiological effects of AcMNPV infection in mammalian cells [18,19,20,21,22], genome-wide transcriptomic changes that occur in mammalian systems have never been investigated. Here, using total RNA-seq analyses, we provide comprehensive transcriptome profiles of both baculovirus and human genes in recombinant baculovirus-infected human cells. In addition, we demonstrate that genes are distinctly regulated by baculovirus promoters and mammalian promoters in our recombinant baculovirus expression system. Our findings provide insights that should facilitate efforts to optimize baculoviral vectors for gene therapy and vaccine development. 

## 2. Materials and Methods 

### 2.1. Cell Culture

Human embryonic kidney 293TT cells (kindly provided by Dr. Schiller, National Cancer Institute, Bethesda, MD, USA) were grown at 37 °C in Dulbecco’s modified Eagle’s medium (DMEM) supplemented with 10% fetal bovine serum (FBS) and 1% penicillin–streptomycin. *Spodoptera frugiperda* 9 (Sf9) (Invitrogen, Carlsbad, CA, USA) insect cells were maintained at 27 °C in Sf-900 medium containing 3% FBS and 1% antibiotic–antimycotic (Gibco, Carlsbad, CA, USA, #15240062) to aid cell growth and to maintain sterile culture conditions. 

### 2.2. Generation of Recombinant Baculoviruses 

A recombinant baculovirus vector expressing HERV*_env_* at the viral surface was constructed by inserting a synthetic codon-optimized envelope gene for HERV type W (GenBank accession number NM014590, GenScript Corp., Piscataway, NJ, USA) into pFastBac1 (Invitrogen) under the control of the polyhedrin (*polh*) promoter (pFastBac-HERV) [23]. Next, the gene encoding enhanced green fluorescent protein (eGFP) was embedded downstream of the CMV promoter in pFastBac-HERV. Recombinant baculoviruses were produced using the Bac-to-Bac baculovirus expression system (Invitrogen) according to the manufacturer’s protocol and were further propagated in Sf9 cells. Virus titer was measured by qRT-PCR using a BacPac qPCR titration kit (Takara Bio, Mountain View, Santa Clara County, CA, USA) and was calculated using the formula: Copies/mL = (raw copy number (copies)) × (1000 μL/mL) × (elution volume (μL))/(sample size (μL)) × (volume (μL) per well).

### 2.3. Infection of Cells with Recombinant Baculoviruses

Both 293TT and Sf9 cells were infected with recombinant AcHERV_env_-CMV-GFP at a multiplicity of infection (MOI) of 30. After 72 h of virus infection, supernatants were removed, and cells were lysed for RNA isolation. 

### 2.4. Fluorescence Microscopy

After 72 h of recombinant baculovirus infection, cells were washed with PBS and fixed with 4% paraformaldehyde for 15 min. Following the washing step, cells were mounted with ProLong™ Gold Antifade Mountant with DAPI (Invitrogen) overnight in the dark. GFP was visualized with a Zeiss LSM 800 confocal microscope using a 40 × oil objective lens. GFP was excited at 488 nm and emission was collected using a 505–530 nm filter. 

### 2.5. RNA Isolation

Total RNA was isolated from recombinant AcHERV*_env_*-CMV-GFP-infected or -uninfected cells using the RNeasy Mini kit (Qiagen, Valencia, CA, USA) according to the manufacturer’s instructions. The quantity and quality of isolated RNA was analyzed using a microplate spectrophotometer (BioTek Instruments, Winooski, VT, USA)

### 2.6. Quantitative Reverse Transcription-polymerase Chain Reaction (RT-qPCR)

cDNA was synthesized from total RNA using SuperScript III first-strand synthesis supermix (Invitrogen), and qPCR was performed using SsoAdvanced Universal SYBR Green Supermix (Bio-Rad, Hercules, CA, USA) on the LightCycler 96 system (Roche, Mannheim, Germany). Gene-specific primers used for qPCR are listed in Table S1. Expression levels of baculovirus genes and immune-response genes were normalized to those of 18s rRNA and glyceraldehyde-3-phosphate dehydrogenase (*GAPDH*), respectively. 

### 2.7. Total RNA-Sequencing (RNA-seq)

Total RNA was isolated from recombinant baculovirus-infected and -uninfected cells and quantified using the PicoGreen (Invitrogen) method using a Victor 3 fluorometer (Perkin Elmer, Norwalk, CT, USA). The integrity of total RNAs was analyzed using a 2100 Bioanalyzer (Agilent Technologies, Palo Alto, CA, USA). RNA samples with an RNA Integrity Number (RIN) value ≥ 7 were used to generate RNA-seq libraries. DNA contamination was removed by treating isolated RNA with DNase, and rRNA was removed using a ribo-zero rRNA removal kit. Purified total RNA was randomly fragmented, synthesized into cDNA, and then used to prepare an RNA-seq library using a TruSeq Stranded Total RNA LT Sample Prep Kit (Illumina, San Diego, CA, USA) according to the manufacturer’s instructions. The quantity and quality of libraries were assessed, and paired-end sequencing with an average length of 100 reads was performed using a NovaSeq 6000 system (Illumina). Three replicates were used for sequencing. The complete raw and normalized Total RNA-seq data have been deposited in the Gene Expression Omnibus (GEO) under accession GSE158603.

### 2.8. Total RNA-seq Data Analysis

Low-quality reads were filtered out by trimming RNA-seq data using Trimmomatic. Subsequent reads were mapped to the reference genome UCSC hg19 or GCF_000838485.1_ViralProj14023 using the Bowtie2 aligner. For comparison of expression levels among all samples, fragments per kilobase of transcript per million mapped reads (FPKM), fold change, and variance were calculated using edgeR. Transcripts with a FPKM > 1 were used for further analysis. Transcripts with a log_2_ fold change ≥ 2 and significant pairwise variance (*p* < 0.05) were classified as differentially expressed genes. Gene set enrichment analysis (GSEA) was performed using the KEGG database and GO term (see the Supplementary Materials). Violin plots, volcano plots, and heat maps were generated in R. 

### 2.9. Statistical Analyses

Gene expression data are presented as means of independent biological replicates with standard error of the mean (s.e.m.). A two-tailed unpaired *t* test was applied for statistical analyses.

## 3. Results

### 3.1. Generation of a Dual Promoter-Controlled Recombinant Baculovirus

**Figure 1:**
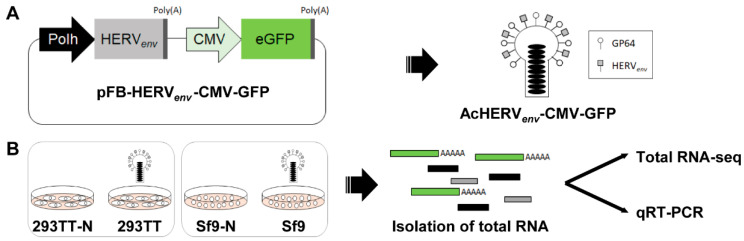
Schematic depiction of the recombinant baculovirus construct and experimental design. (**A**) A pFastBac-HERV*_env_*-CMV-GFP plasmid was constructed to express the envelope protein HERV*_env_* and GFP under the control of the baculovirus *polh* promoter and human CMV promoter, respectively. The recombinant baculovirus, AcHERV*_env_*-CMV-GFP, was generated using the Bac-to-Bac baculovirus expression system and was propagated in Sf9 cells. (**B**) Human embryonic kidney 293TT cells and insect Sf9 cells were infected with AcHERV*_env_*-CMV-GFP at a MOI of 30. Gene expression profiles were obtained by performing total RNA-seq and qRT-PCR analyses using total RNA isolated 72 h after virus infection. 293TT-N, uninfected 293TT cells; 293TT, infected 293TT cells; Sf9-N, uninfected Sf9 cells; Sf9, infected Sf9 cells.

A recombinant baculovirus that is regulated by both a baculovirus promoter and a mammalian promoter was used to examine the impact of baculovirus infection on genome-wide transcriptome profiles in mammalian cells. In our previous study, we established the recombinant baculovirus AcHERV*_env_* expressing the envelope protein of human endogenous retrovirus (HERV) at the viral surface, which enhances virus infectivity in mammalian cells [23,24,25]. HERV*_env_* is embedded downstream of the baculovirus polyhedrin (*polh*) promoter. To visualize mammalian promoter-regulated gene expression, we further inserted the GFP gene under the control of the *Cytomegalovirus* (*CMV*) promoter, yielding pFB-HERV*_env_*-CMV-GFP (Figure 1A). The recombinant baculovirus, AcHERV*_env_*-CMV-GFP, was produced from pFB-HERV*_env_*-CMV-GFP using the Bac-to-Bac baculovirus expression system and was further propagated in Sf9 cells. For preparation of total RNA-seq samples, human 293TT and insect Sf9 cells were infected with AcHERV*_env_*-CMV-GFP at a MOI of 30 for 72 h (Figure 1B), and total RNA was isolated. 

**Figure 2:**
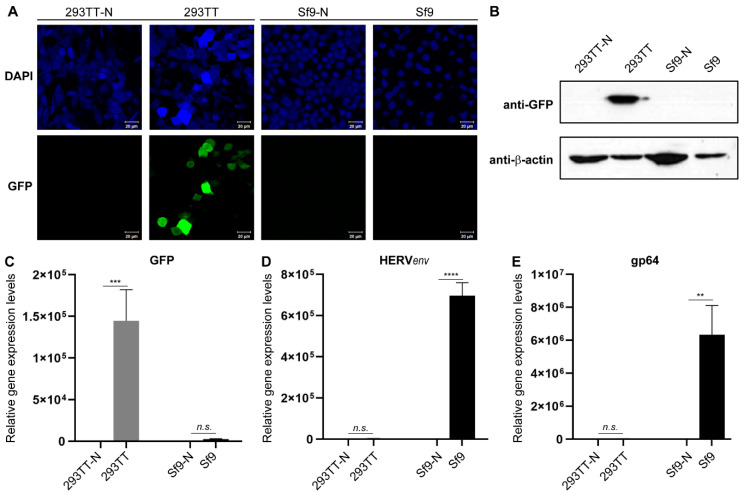
Expression levels of representative genes controlled by mammalian and baculovirus promoters. (**A**) Fluorescence images of CMV promoter-regulated GFP expression in AcHERV*_env_*-CMV-GFP-infected 293TT and Sf9 cells. 293TT-N and Sf9-N were used as uninfected controls, respectively. DAPI visualizes nuclei. Scale bars in all fluorescence images, 20 μm. (**B**) Western blot analyses of GFP expression in AcHERV*_env_*-CMV-GFP-infected 293TT and Sf9 cells. The levels of GFP protein were assayed using anti-GFP antibody and normalized relative to β-actin levels, used a loading control. qRT-PCR analyses of (**C**) GFP, (**D**) baculovirus promoter-regulated HERV*_env_*, and (**E**) gp64 expression in AcHERV*_env_*-CMV-GFP-infected 293TT and Sf9 cells. GFP, HERV*_env_*, and gp64 RNA levels were normalized to 18s rRNA levels. Results are shown as the means ± s.e.m. of six independent replicates. ** *p* < 0.001, *** *p* < 0.0001, **** *p* < 0.00001. *n.s*., not significant.

To assess distinct gene regulatory events controlled by mammalian and baculoviral promoters, GFP signals were visualized by fluorescence microscopy and Western blotting and qRT-PCR analyses were performed (Figure 2 and Figure S1). As expected, green fluorescence and GFP expression was only detected in AcHERV*_env_*-CMV-GFP-infected 293TT cells, and not in Sf9 cells or uninfected controls (Figure 2A–C). Next, we examined expression levels of *gp64* and the gene encoding the viral surface protein HERV*_env_*, which are controlled by baculoviral promoters (Figure 2D,E). Both HERV*_env_* and *gp64* were prominently expressed in AcHERV*_env_*-CMV-GFP-infected Sf9 cells, but not in virus-infected 293TT cells or uninfected controls. These results indicate that the recombinant AcHERV*_env_*-CMV-GFP baculovirus expression system ensures promoter-specific gene regulation. 

### 3.2. Comparative Baculovirus Transcriptome Profiles in Insect and Human Cells

**Figure 3:**
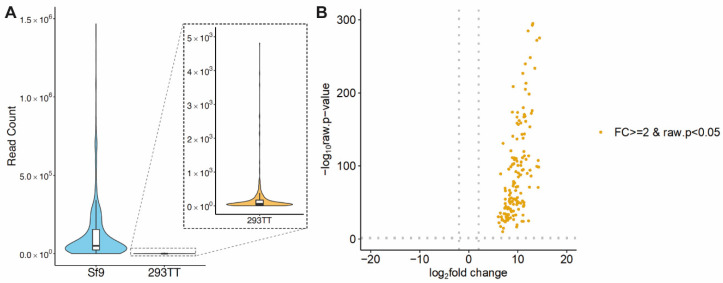
Comparative baculovirus transcriptome profiles of recombinant baculovirus-infected human and insect cells. (**A**) Violin plots illustrate read counts obtained for AcHERV*_env_*-CMV-GFP-infected 293TT and Sf9 cells. (**B**) Volcano plot shows the fold change (log_2_ transformed) and variance for all transcripts in AcHERV*_env_*-CMV-GFP-infected 293TT cells relative to virus-infected Sf9 cells. Differentially expressed transcripts are highlighted in orange. Dotted gray lines indicate two-fold changes and a *p*-value ≤ 0.05.

To obtain genome-wide baculovirus transcriptome profiles, we conducted total RNA-seq analyses on AcHERV*_env_*-CMV-GFP-infected 293TT and Sf9 cells. Total RNA-seq reads were mapped to *A. californica* nuclear polyhedrosis virus (*AcNPV*, GCF_000838485.1_ViralProj14023). These analyses showed that 148 baculovirus genes were associated with AcHERV*_env_*-CMV-GFP-infected 293TT cell transcripts, and 150 genes were associated with AcHERV*_env_*-CMV-GFP-infected Sf9 cell transcripts. Two genes that were not expressed in 293TT cells were *Orf20* and *Orf160*. *Orf20* is involved in actin rearrangement and the enhancement of viral movement [26]. The biological function of *Orf160* has not yet been determined. No baculovirus genes were linked to uninfected 293TT cell transcripts. Notably, the mapping ratio of virus-infected 293TT cell transcripts was less than 0.1%, whereas a relatively high mapping ratio (>46%) was found for virus-infected Sf9 cell transcripts (Table 1). These data suggest that baculovirus infection of 293TT cells induces only basal levels of viral gene expression. Using independent biological replicates, we confirmed the reproducibility of RNA-seq data (Figure S2A). Gene expression profiles were distinct between virus-infected 293TT and Sf9 experimental groups (Figure S2B). Overall, baculovirus genes were rarely expressed in AcHERV*_env_*-CMV-GFP-infected 293TT cells; however, some genes were still expressed at modest levels. To further understand these gene sets, we analyzed total read counts obtained from virus-infected 293TT and Sf9 cells. Mean read counts of baculovirus genes from virus-infected Sf9 cells (133,635 read counts) were more than 700-fold higher than those from 293TT cells (180 read counts) (Figure 3A). *PE38*, a baculovirus early expressed gene, showed the highest read count (3370) in virus-infected 293TT cells, whereas the baculovirus late-expressed gene *39K/pp31* showed the highest read count (1,268,493) in infected Sf9 cells. Expression levels of all 148 baculovirus genes associated with AcHERV*_env_*-CMV-GFP-infected 293TT cell transcripts were exclusively and significantly increased by more than two-fold (log2 transformed) in virus-infected Sf9 cells compared with virus-infected 293TT cells (Figure 3B).

**Figure 4:**
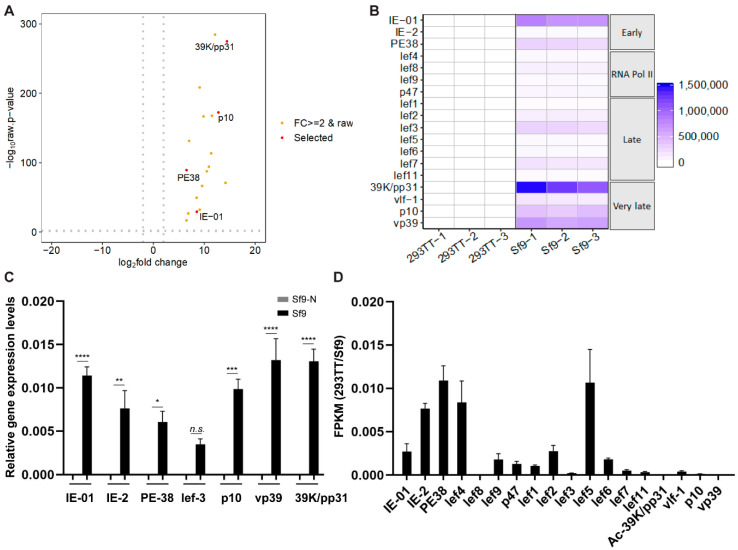
Transcriptome profiles of baculovirus early- and late-expressed genes in human cells. (**A**) Volcano plot shows the fold change (log_2_ transformed) and variance for baculovirus early- and late-expressed genes in AcHERV*_env_*-CMV-GFP-infected 293TT cells relative to virus-infected Sf9 cells. (**B**) Heatmap showing the relative changes in expression of representative early and late-expressed baculovirus genes. (**C**) qRT-PCR analysis of baculovirus early and late genes in Sf9 cells, confirming the RNA-seq data. Results are shown as the means ± s.e.m. of six independent replicates. * *p* < 0.05, ** *p* < 0.001, *** *p* < 0.0001, **** *p* < 0.00001. *n.s.*, not significant. (**D**) Bar graph showing relative levels of expression of 18 representative baculovirus genes in 293TT cells. FPKM values of baculovirus gene transcript in 293TT cells were normalized to those of Sf9 cells.

To further understand the baculovirus transcriptome profiles of virus-infected 293TT and Sf9 cells, we selected 18 baculovirus genes that have well-known functions [7,8,9,10,27,28,29,30] and compared expression levels of these genes between virus-infected 293TT cells and Sf9 cells. All 18 genes showed a more than two-fold change in expression in infected Sf9 cells compared with that in 293TT cells, changes that were statistically significant (Figure 4A). Among these 18 baculovirus genes, the baculovirus late-expressed genes *39K/pp31* and *p10* showed greater differential expression (14- and 13-fold change) than the early-expressed genes *PE38* and *IE-01* (6- and 9-fold change). In agreement with the RNA-seq data, qRT-PCR results showed that overall baculovirus gene expression levels were significantly lower in virus-infected 293TT cells than in virus-infected Sf9 cells (Figure S3). Overall, baculovirus very late-expressed genes, including *39K/pp31*, *p10*, and *vp39*, were dramatically induced in virus-infected Sf9 cells relative to 293TT cells (Figure 4B,C). RNA polymerase II subunits, including *lef-4*, *lef-8*, *lef-9*, and *p47* [31], were modestly increased in Sf9 cells. Notably, expression levels of early-expressed genes such as *PE38*, *IE-01*, and *IE-02* were higher than those of late-expressed genes in 293TT cells (Figure 4D). In comparison, expression levels of very late-expressed genes such as *39K/pp31*, *p10*, and *vp39* were higher than those of early expressed genes in Sf9 cells. These data suggest that, although recombinant baculovirus infection of human cells can induce basal levels of baculovirus early gene expression, rare expression of baculovirus late genes may not be sufficient to lead to the assembly of mature viruses and further virus amplification. 

### 3.3. Induction of Immune-Response Genes upon Baculovirus Infection in Human Cells

**Figure 5:**
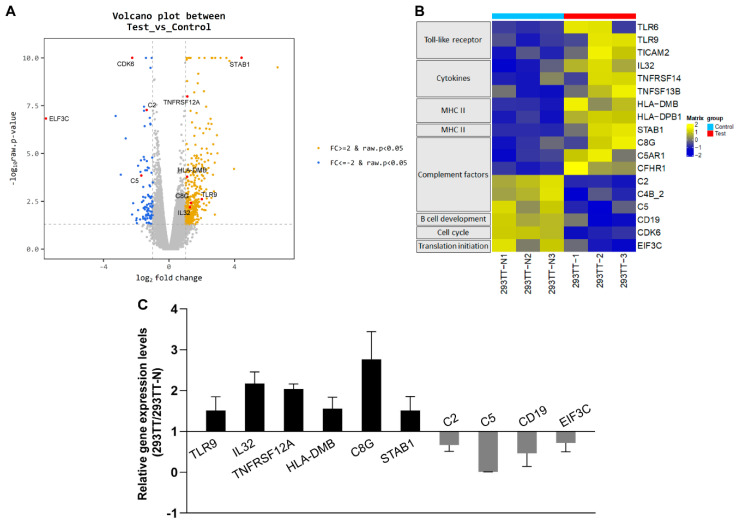
Transcriptome profiles of host immune-response genes. (**A**) Volcano plot shows the fold change (log_2_ transformed) and variance for human gene transcripts in AcHERV*_env_*-CMV-GFP-infected 293TT cells relative to uninfected controls. Differentially expressed transcripts are highlighted in orange. (**B**) Heatmap depicting relative change in the expression of host immune-response genes. (**C**) qRT-PCR analysis of the indicated genes in AcHERV*_env_*-CMV-GFP-infected 293TT cells and uninfected controls verifies the RNA-seq data. mRNA levels of indicated genes in 293TT and 293TT-N were normalized to *GAPDH* levels. Gene expression levels of 293TT relative to 293TT-N are shown as the means ± s.e.m. of six independent replicates.

Several lines of evidence have shown that baculovirus infection can elicit immune responses in human cells [11,20,32]. To further analyze the genome-wide impact of baculovirus infection on human gene expression, we mapped the total RNA-seq reads obtained from infected and uninfected 293TT cells to the human reference genome. The mapping ratio was over 95% in both experimental groups (Table S2). To investigate whether baculovirus infection affects the human transcriptome profile, we carried out differential gene expression analyses on RNA-seq data from infected 293TT cell cultures and uninfected controls. Of 16,883 human genes, 440 showed differential gene expression greater than two-fold (log_2_ transformed, *p* < 0.05); 347 genes were upregulated and 93 genes were downregulated in infected 293TT cultures compared with uninfected controls (Figure 5A). 

Gene set enrichment analysis (GSEA) of RNA-seq data have demonstrated that genes associated with immune responses, including those involved in the Toll-like receptor (TLR) signaling pathway, cytokine–cytokine receptor interaction, complement and coagulation cascades, and cell adhesion molecules, were significantly induced in AcHERV*_env_*-CMV-GFP-infected 293TT cells compared with uninfected controls (Figure 5B and Figure S4). In the TLR signaling pathway, expression levels of *TLR6*, *TRL9*, and *TICAM2* genes were increased by more than two-fold relative to uninfected controls. In the cytokine–cytokine receptor interaction pathway, *IL32*, *TNFRSF14*, and *TNFRSF13B* levels were induced more than two-fold. Among MHC II molecules, *HLA-DMB* and *HLA-DPB1* levels were increased. In complement and coagulation cascades, *C8G*, *C5AR1*, and *CFHR1* exhibited significant gene induction. Among cell adhesion molecules, *STAB1* showed the elevated expression levels. Several complement components (*C2*, *C4B_2*, and *C5*) and factors involved in B cell development (*CD19*), the cell cycle (*CDK6*), and translation initiation (*EIF3C*) were downregulated in virus infected 293TT cells. Changes in the expression of representative genes, including *TLR9*, *IL32*, *TNFRSF12A*, *HLA-DMB*, *C8G*, *STAB1*, *C2*, *C5*, *CD19*, and *EIF3C*, were validated by qRT-PCR analyses (Figure 5C). Consistent with previous reports, these data demonstrate that baculovirus infection induces some immune response genes in mammalian cells. Our data further provide extended transcriptome profiles that may deepen our understanding of the genome-wide impact of baculovirus infection in mammals. 

## 4. Discussion

Recent advances in next-generation sequencing technologies have led to a better understanding of various genomic structures of viruses and the host transcriptomic changes that occur upon infection with a specific virus. The genomic structure of the baculovirus AcMNPV [33] and the AcMNPV transcriptome in the Ni insect cell line [34] have been previously identified. However, the biological impact of AcMNPV infection in mammals has never been demonstrated at the genome-wide level. To compare baculovirus transcriptome profiles in insect and mammalian cells, we conducted total RNA-seq analyses on both insect Sf9 cells and human 293TT cells after infection with recombinant baculovirus. We first examined whether the dual promoter system in our recombinant baculovirus distinctly regulated genes specific to each promoter. As expected, expression of GFP was restricted to AcHERV*_env_*-CMV-GFP-infected 293TT cells, indicating tight regulation of the mammalian CMV promoter. In contrast, levels of the viral surface proteins HERV*_env_* and *gp64* were only dramatically induced in Sf9 cells under the control of baculovirus promoters. 

We further mined our RNA-seq data to determine whether any baculovirus genes were induced in mammalian cells. We found that most baculovirus genes were expressed at basal levels in virus-infected 293TT cells, although some genes showed modest expression. Baculovirus early-expressed genes, including *PE38*, *IE-01*, and *IE-2*, were slightly upregulated, whereas late genes essential for virus assembly were relatively silent. qRT-PCR analysis showed that, 24 h after infection of human HepG2 and 293 cells, AcMNPV could transcribe baculovirus immediate early genes, *IE-1* and *IE-2* [19]. RT-PCR analysis showed that a second widely used baculovirus species, *Bombyx Mori* nucleopolyhedrovirus *(BmNPV)*, could induce the expression of baculovirus early genes, *IE-1*, *he65*, and *IE-0*, but not late genes, such as *p39* and *pol*, in 293 and Schwann cells [35]. In agreement with previous reports, our global RNA-seq results also revealed that baculovirus early genes were modestly expressed in human 293TT cells. In comparison, baculovirus late-expressed genes, including *p10* and *39K/pp31*, were highly expressed in baculovirus-producing Sf9 cells relative to early-expressed genes. Baculovirus early genes are typically expressed until ~6 h post infection (h.p.i.) and are essential for viral DNA synthesis and the expression of late genes [7,8]. The late genes (~6–18 h.p.i.) and very late genes (~18–72 h.p.i.) are crucial for transcription of the viral structural proteins required for virus assembly [9,10]. Our RNA-seq data suggest that the limited expression of baculovirus early genes in 293TT cells may not be sufficient for the full induction of late gene expression and viral DNA synthesis and may further interfere with intact virus assembly and virus budding.

Finally, we explored the genome-wide impact of baculovirus infection on the human transcriptome. A previous microarray analysis showed that infection of 293 cells by *BmNPV* did not appreciably alter the expression of human genes, but did induce small changes in expression of 22 genes [35]. Our RNA-seq results showed that some immune response genes were sensitive to recombinant *AcMNPV* infection and were slightly upregulated in virus-infected 293TT cells. These included genes involved in the TLR signaling pathway or cytokine–cytokine receptor interaction, or those encoding complement factors or adhesion molecules. Previous studies have reported that some inflammatory cytokines, including type I interferons, interleukins, and tumor necrosis factors, can be induced upon baculovirus infection in mammalian cells and in mice [11,18,20,21,32,36]. Activation of T cell responses through MHC epitopes and induction of the complement system have also been reported [15,16,17]. Viral vector-mediated gene delivery was expected to induce immune responses. The most widely used AAV vector has been shown to induce both innate and adaptive immune responses [37,38,39]. Similar to our results on baculovirus, AAV activates the TLR9 signaling pathway. However, AAV further induces NF-κb, interferon response genes, and genes encoding several proinflammatory cytokines, including TNF-α, IL-6, CCL5, MCP-1, and type I IFNs, as well as activating CD8+ T cells. Although direct comparisons are necessary, these findings suggest that baculoviral vectors may be the safer choice for gene delivery systems, as they induce only limited changes in host innate immunity. Our RNA-seq data also reveal that few genes involved in B cell development, complement, cell cycle, and translation initiation were downregulated in virus-infected 293TT cells. The induction of immune responses may lead to further inhibition of protein translation and cell division of virus infected cells, thereby protecting the host. Our data verify the immunogenic features of recombinant baculovirus infection at the genome-wide level. These findings may be useful for efforts to enhance host immunity as part of an effective vaccine strategy. Collectively, the results of our study expand our understanding of transcriptomic changes that occur upon baculovirus infection in both human and insect cells. These data should provide a framework for the design of an advanced baculovirus expression system and help ensure its safety for gene delivery, vaccine development and clinical therapeutics.

## 5. Conclusions

With the advent of RNA-seq protocols, extensive transcriptome landscapes have been uncovered in diverse species and specific biological environments. In this study, we unraveled the genome-wide impact of recombinant baculovirus infection in both insect and human cells. Our data clearly show that baculovirus infection generates different baculovirus transcriptomes in invertebrate and vertebrate cells and affects the human transcriptome. Further genome-wide analyses of baculovirus-infected living organisms will deepen our understanding of the efficacy and safety of baculovirus expression systems for clinical use. 

## Figures and Tables

**Table 1:** Mapping ratio of baculovirus genes for AcHERV*_env_*-CMV-GFP-infected cells.

Sample	Sample No.	Number of Processed Reads	Number of Mapped Reads
293TT-N	1	123,252,764	0 (0.00%)
	2	130,201,768	16 (0.00%)
	3	126,360,742	0 (0.00%)
293TT	1	100,237,542	56,316 (0.06%)
	2	98,908,778	107,364 (0.11%)
	3	148,781,606	170,092 (0.11%)
Sf9	1	136,870,040	63,122,142 (46.12%)
	2	127,920,774	56,971,000 (44.54%)
	3	112,371,568	51,938,850 (46.22%)

## Supplementary Materials

The following are available online at https://www.mdpi.com/2073-4425/11/11/1306/s1, Figure S1: Bright field microscope images of uninfected and AcHERV*_env_*-CMV-GFP infected 293TT and Sf9 cells, Figure S2: Assessment of intra- and intergroup variability, Figure S3: qRT-PCR analysis of baculovirus early and late gene expression levels in recombinant baculovirus infected 293TT and Sf9 cells, Figure S4: KEGG pathway analyses, Table S1: Gene-specific primer sequences for qRT-PCR analyses, Table S2: Mapping ratio of human genes for recombinant baculovirus infected and uninfected cells.
