# Supplementary material for: Unraveling the Genome-Wide Impact of Recombinant Baculovirus Infection in Mammalian Cells for Gene Delivery
Source: Genes (Basel). 2020 Nov 4;11(11):1306. doi: 10.3390/genes11111306 (PMC7694231; doi:10.3390/genes11111306)
Supplement: Supplementary file 1 [file genes-11-01306-s001.zip › genes-991593-supplementary/genes-991593-Supplementary Figures and Tables .docx]

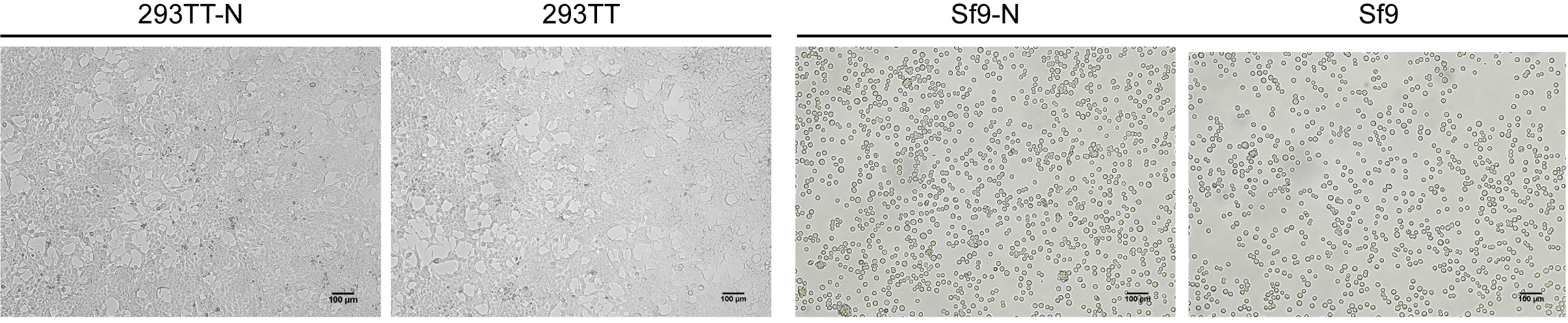


**Figure S1.** Bright-field microscopy images of uninfected and AcHERV*_env_*-CMV-GFP–infected 293TT and Sf9 cells. A cytopathic effect was observed only in infected Sf9 cells. Cell images were captured using a Nikon Ts2R-PHFL inverted microscope (20 ×). 293TT-N, uninfected 293TT cells; 293TT, infected 293TT cells; Sf9-N, uninfected Sf9 cells; Sf9, infected Sf9 cells. Scale bars in all images, 100 mm.


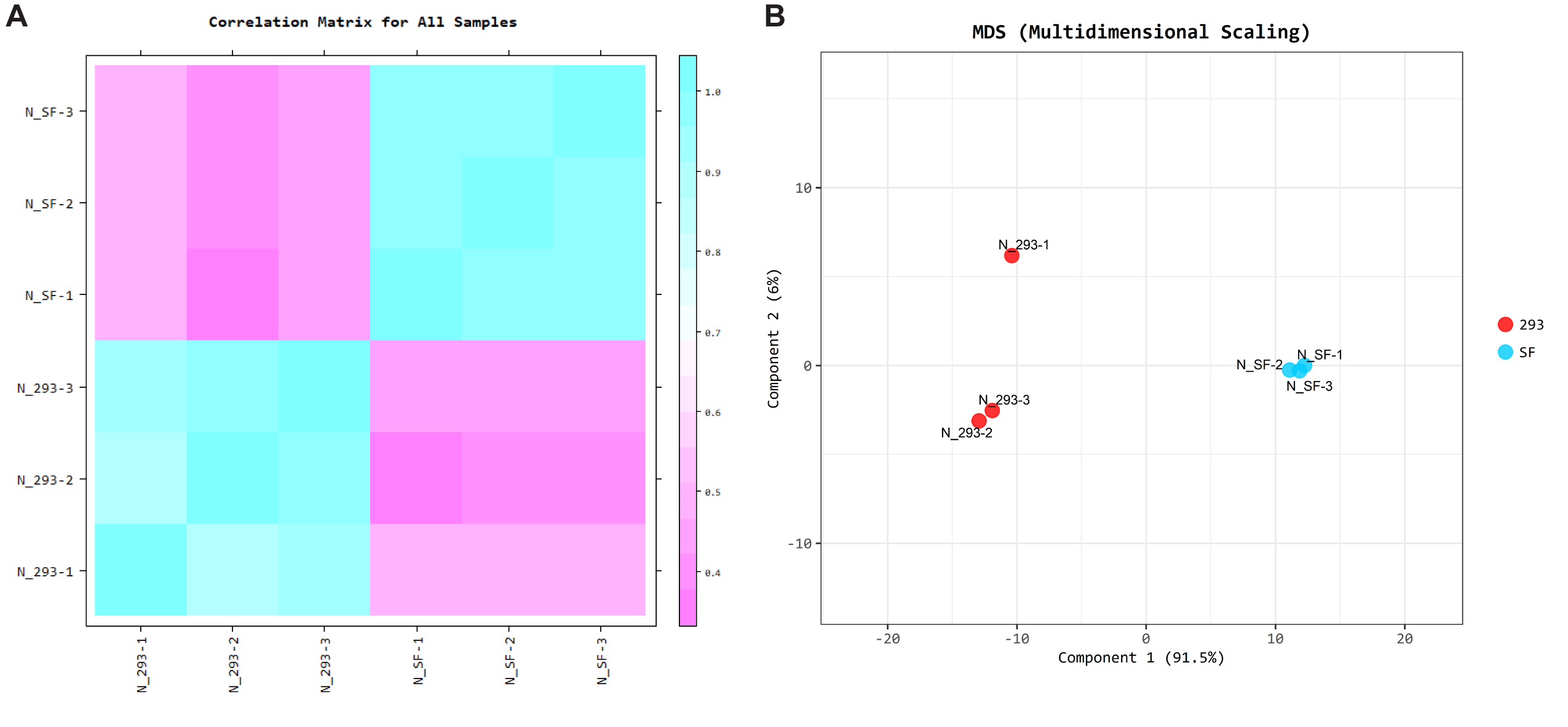


**Figure S2.** Assessment of intra- and intergroup variability. (**A**) Pearson’s correlation plot displaying correlations between samples. N-293, virus-infected 293TT cells; N-SF, virus-infected Sf9 cells. Scale bar represents the range of correlation coefficients. (**B**) Multidimensional scaling (MDS) plot depicting the levels of similarity for individual cases in an experimental group.


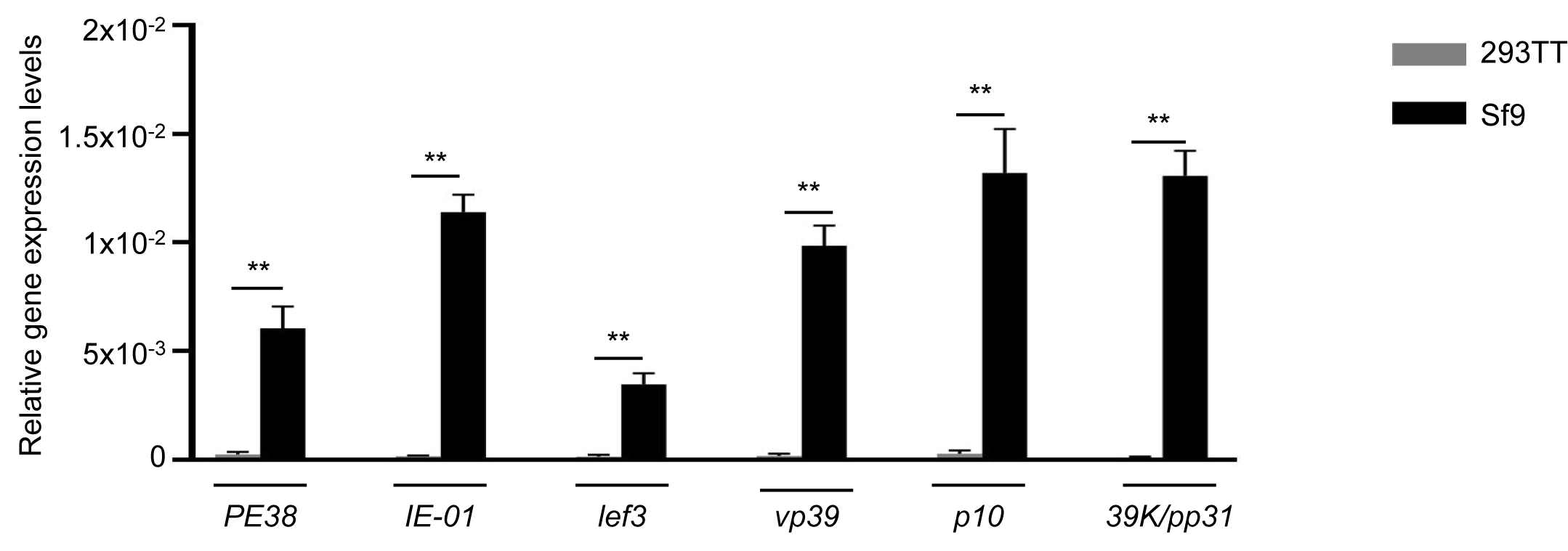


**Figure S3.** qRT-PCR analysis of baculovirus early- and late-expressed gene levels in recombinant baculovirus-infected 293TT and Sf9 cells. Results are shown as the means ± s.e.m. of six independent biological replicates. A two-tailed unpaired *t*-test was used to evaluate the significance of differences between AcHERV*_env_*-CMV-GFP–infected 293TT cells and Sf9 cells (**, *p* < 0.001).


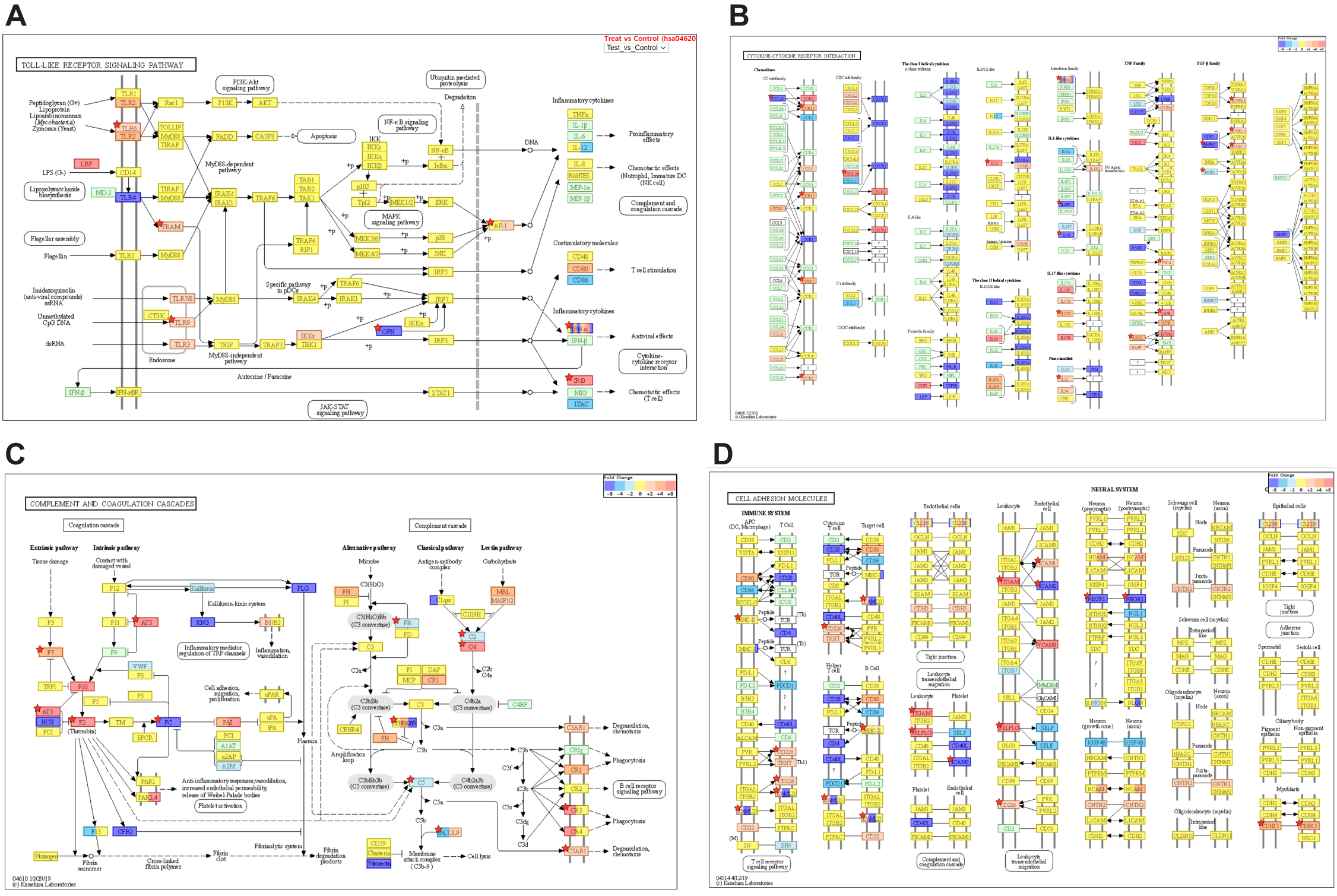


**Figure S4.** KEGG pathway analyses: (**A**) Toll-like receptor signaling pathway; (**B**) cytokine–cytokine receptor interaction; (**C**) coagulation network; and (**D**) cell adhesion molecules. Scale bar indicates the fold change (log_2_ transformed) of gene expression levels, and the red star indicates genes induced more than two-fold in infected 293TT cultures relative to uninfected controls.

**Table S1.** Gene-specific primer sequences for qRT-PCR analyses.

| **Target gene** | **Primer Sequences** | |
| --- | --- | --- |
| *GFP* | Forward | 5’–GTGAACCGCATCGAGCTGAAGGG–3’ |
|  | Reverse | 5’–GTTCTGCTGGTAGTGGTCGGCGA–3’ |
| HERV*_env_* | Forward | 5’–GATCAGCCAGCTGACAAGGGTGCA–3’ |
|  | Reverse | 5’–TAAGGCCGGAAATTCAGTGGCAGG–3’ |
| *gp64* | Forward | BacPAK qPCR Titration Kit (Takara, #631415) |
|  | Reverse | 5’–AAAAAAAAAAAAAAAAAAAA–3’ |
| *IE-01* | Forward | 5’–GAATCCCTTGAGCAGCCTGTTG–3’ |
|  | Reverse | 5’–GTTGCACAGCTTTGAATTGTGCT–3’ |
| *PE38* | Forward | 5’–AGAACGGAGCGTGATTAGTGTCG–3’ |
|  | Reverse | 5’–ATGTCCTCACCTGATGCTGCAACT–3’ |
| *lef3* | Forward | 5’–GCGACGAGTTGAACATTGCCGA–3’ |
|  | Reverse | 5’–CTCGCCGTTCTCGACAGTTACGTA–3’ |
| *39K/pp31* | Forward | 5’–TGAAGGAGAGCTGCGAAATGC–3’ |
|  | Reverse | 5’–CACGTACTCTGTAGTAGACGGTG–3’ |
| *p10* | Forward | 5’–AACTCAGTTAAACGGGCTGGA–3’ |
|  | Reverse | 5’–CTTGGAACTGCGTTTACCACGAC–3’ |
| *Vp39* | Forward | 5’–GACACCACAAACCCGAACACGT–3’ |
|  | Reverse | 5’–TGCAAGTACTCGGGCGCTACTG–3’ |
| *TLR9* | Forward | 5’–AGTCCTCGACCTGGCAGGAA–3’ |
|  | Reverse | 5’–GCGTTGGCGCTAAGGTTGA–3’ |
| *IL32* | Forward | 5’–TCAAAGAGGGCTACCTGGAGAC–3’ |
|  | Reverse | 5’–TCTGTTGCCTCGGCACCGTAAT–3’ |
| *TNFRSF12A* | Forward | 5’–GCTCGCCCACTCATCATTCATTC–3’ |
|  | Reverse | 5’–TCTGTTCTGGAGCCAGAGGCA–3’ |
| *C8G* | Forward | 5’–TCCTGGGACTTGGTGGTGCTAC–3’ |
|  | Reverse | 5’–TTGGGCTGGATGGTGCTGATG–3’ |
| *HLA-DMB* | Forward | 5’–CACTTACACCTGTGTGGTAGAGC–3’ |
|  | Reverse | 5’–GCAGACACAGAAACCTTCAGGG–3’ |
| *STAB1* | Forward | 5’–GAACCATGTGCCACTGGAAGGC–3’ |
|  | Reverse | 5’–AGCGGAATCTCCTGGTGCAGTT–3’ |

**Table S2.** Mapping ratio of human genes for recombinant baculovirus-infected and -uninfected cells.

| **Sample** | **Sample No.** | **Number of Processed Reads** | **Number of Mapped Reads** |
| --- | --- | --- | --- |
| 293TT-N | 1 | 100,237,542 | 94,887,334 (94.66%) |
|  | 2 | 98,908,778 | 94,449,046 (95.49%) |
|  | 3 | 148,781,606 | 143,852,593 (96.69%) |
| 293TT | 1 | 123,252,764 | 119,002,441 (96.55%) |
|  | 2 | 130,201,768 | 125,571,010 (96.44%) |
|  | 3 | 126,360,742 | 121,443,479 (96.11%) |
